# Ethnomedicinal Knowledge of *Trichocentrum ascendens* (Orchidaceae) in an Indigenous Region of Oaxaca, Mexico

**DOI:** 10.3390/plants15060873

**Published:** 2026-03-12

**Authors:** Mayra Ariadna García-Patiño, Luicita Lagunez-Rivera, Gabriela Soledad Barragán-Zárate, Jesús Alejandro Ríos-Solis, Rodolfo Solano

**Affiliations:** Laboratorio de Extracción y Análisis de Productos Naturales, Centro Interdisciplinario de Investigación para el Desarrollo Integral Regional Unidad Oaxaca, Instituto Politécnico Nacional, Hornos 1003, Santa Cruz Xoxocotlán 71233, Oaxaca, Mexico; mgarciap2200@alumno.ipn (M.A.G.-P.); jrioss2200@alumno.ipn (J.A.R.-S.)

**Keywords:** cultural diseases, medicinal plants, orchids, traditional knowledge, traditional medicine

## Abstract

*Trichocentrum ascendens*, commonly known as “*cola de rata*”, is an orchid traditionally used by Indigenous communities to remove warts and heal wounds and in cultural cleansing rituals (“*limpia*”). However, additional medicinal uses preserved by the Chinantec people of Oaxaca, Mexico, remain largely undocumented and are at risk of disappearing, as this knowledge is now held by only a few individuals. This study gathered information on the therapeutic applications of *T. ascendens* in the Chinantla region through semi-structured interviews with key collaborators. Information was collected regarding the socio-demographic profiles of the collaborators, as well as the conditions treated, plant parts used, preparation modes, and doses. The species is used to alleviate headaches, toothaches, stomach pains, menstrual pains, body aches, kidney diseases, and inflammation, as well as to treat cultural diseases known as “*mal de orin*” and “*sangre sucia*”. Infusions made from mature or developing leaves, along with topical application of crushed leaves, are the main forms of administration. The traditional knowledge documented here underscores the importance of further research to identify the bioactive compounds in *T. ascendens* and to evaluate their potential inflammatory and analgesic effect. Such studies could lead to the discovery of new pharmacologically active molecules while preserving valuable traditional knowledge.

## 1. Introduction

The vascular flora of Mexico comprises over 23,000 species [1], of which 4500 have been reported to have some medicinal uses [2]. Indigenous communities preserve traditional and local knowledge about medicinal plants [3,4], passed down through generations, often for centuries [5]. Oaxaca, the Mexican state with the greatest floristic diversity [1], is home to numerous indigenous peoples and a vast reservoir of knowledge about its flora [6]. Among this rich botanical heritage, medicinal plants are distinguished with over 1000 species documented [7]. The Chinantec people, one of the ethnic groups of Oaxaca, predominantly inhabit the northern region of the state, known as Chinantla, which is home to 1021 vascular plant species, with Orchidaceae being the third most diverse family [8]. Orchids have been used therapeutically for thousands of years and remain an important part of traditional medicine in various regions around the world [9,10,11]. The orchid genus *Trichocentrum* Poepp. & Endl., which includes 85 species such as *Cohniella* Pfitzer, *Lophiaris* Raf., and *Lophiarella* Szlach., is found throughout the American tropics [12,13,14]. It has a center of diversity in Mexico, with 29 recorded species. Three species in this genus have reported medicinal uses, all native to Mexico: *Trichocentrum ascendens* (Lindl.) M.W. Chase & N.H. Williams, *Trichocentrum pachyphyllum* (Hook.) R. Jiménez & Carnevali and *T. cebolleta* (Jacq.) M.W. Chase & N.H. Williams.

The use of *Trichocentrum cebolleta* was reported in Colombia, where its pseudobulbs, crushed with salt, are applied externally to treat severe bruises, bone fractures, and wounds [15]. Since the species has reduced pseudobulbs, the leaves are the most prominent feature and are used to treat foot infections, wounds, heart pain, and abdominal colic [16,17]. On the other hand, it has been documented that the Raramuri (Tarahumaras), an ethnic group in northern Mexico, mainly from Chihuahua, use the leaves of this species in religious rituals as a substitute for peyote, *Lophophora williamsii* (Lem. ex J.F. Cels) J.M. Coult., when it is scarce in the region [16,18]. However, within Mexico, the distribution of *T. cebolleta* is restricted to the Yucatan Peninsula. On the Pacific side of the country, from Sonora and Chihuahua to Oaxaca, a similar species, *Trichocentrum brachyphyllum* (Lindl.) R. Jiménez [19], is found instead. It is likely that the medicinal and religious uses attributed to *T. cebolleta* in these regions actually correspond to *T. brachyphyllum*. This orchid contains phenanthrenes, dihydrophenanthrenes, and unidentified alkaloids [18], some of which exhibit hallucinogenic properties. Furthermore, *T. brachyphyllum* exhibits a concentration-dependent vasorelaxant effect [20]. *Trichocentrum pachyphyllum*, commonly known as “*oreja de burro*” (donkey ear) because of the shape of its leaves, is reportedly used to treat arterial hypertension [21,22].

**Figure 1:**
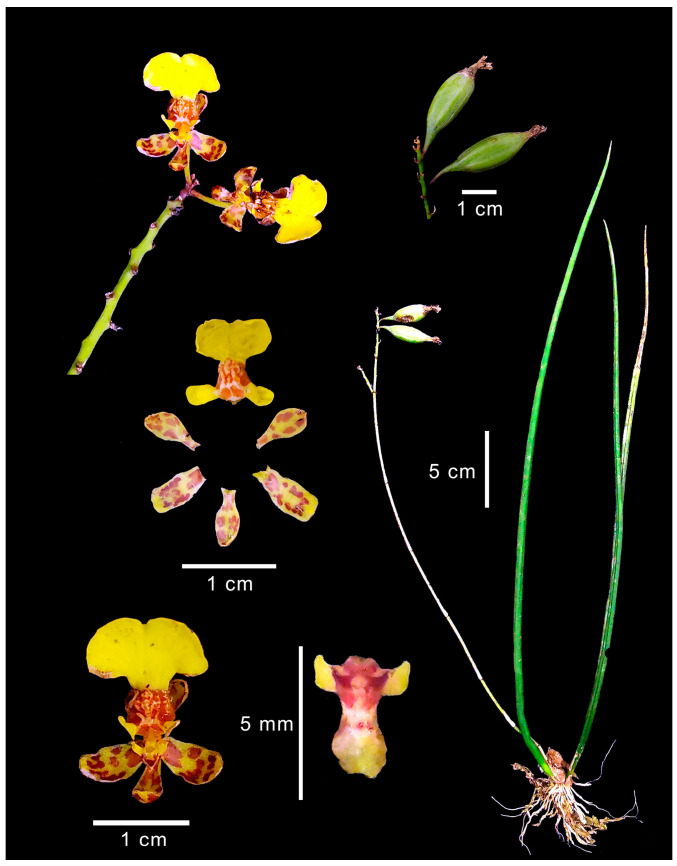
*Trichocentrum ascendens* (Lindl.) M.W, Chase & N.H. Williams. Photographic composition by R. Solano based on M. García s.n.

*Trichocentrum ascendens* (Figure 1), known as “*cuerno de chivo*” (goat horn) [23] or “*cola de rata*” (rat tail) [19], is named for its large, fleshy, and cylindrical leaves [24,25]. This species ranges from Mexico to Colombia and Venezuela [12,13,14]. In Mexico, it is found in Campeche, Chiapas, Oaxaca, Quintana Roo, Tabasco, Tamaulipas, Veracruz, and Yucatán [1,14,26]. Medicinal uses for *T. ascendens* have been documented in Yucatan for removing warts [27], in Veracruz for wound healing, and in the cultural cleansing ritual known as “*Limpia*” [17,28]. The “*Limpia*” involves sweeping the body with aromatic plants to heal ailments considered psychosomatic [29,30].

Very few chemical studies have been conducted on *T. ascendens*; only certain pigments, such as carotenoids (neoxanthin, lutein, and chlorophyll) and xanthophylls (violaxanthin, anteraxanthin, and zeaxanthin), have been identified [31]. However, no pharmacological studies have validated its traditional medicinal uses. The limited knowledge about this orchid may result from the absence of systematic documentation of its medicinal uses, which constrains the exploration of its potential biological activities and pharmacologically relevant secondary metabolites.

This study aimed to document the traditional knowledge and therapeutic practices associated with the use of *T. ascendens* in the Chinantla region of Oaxaca, Mexico, through semi-structured interviews with key collaborators.

## 2. Results

### 2.1. Socioeconomic Aspects of the Collaborators

Table 1 summarizes the socioeconomic data of the seven individuals identified as key collaborators and interviewed in the study area, all of whom possess knowledge about the medicinal use of *Trichocentrum ascendens*. Of these, 57% were women while 43% were men; all were adults aged between 29 and 59, residing in five communities within the study area: La Gran Lucha (municipality Valle Nacional), Plan de Aguila (municipality San Juan Chiltepec), Chiltepec (municipality San José Chiltepec), Ejido Emiliano Zapata (municipality Santa María Jacatepec), and Jacatepec (municipality Santa María Jacatepec).

Regarding education levels, 14% of the participants had no formal schooling, 14% had completed elementary school, 28% had a high school education, and 44% were undergraduate students. Among the women, 75% were engaged in traditional medicine as their primary economic activity, either as medicinal plant traders or as healers, and the remaining 25% worked as vendors of artisanal and natural products at cultural and gastronomic fairs. Among the men, 67% were engaged in traditional medicine as their main economic activity, while the remaining 33% were farmers. In terms of language, 28% of key collaborators speak only Spanish, while 72% speak Spanish and the native language of the region, Chinanteco. Regarding the medicinal knowledge of *T. ascendens*, 14% female collaborators learned it from a traditional healer, whereas the remaining collaborators, both male and female, learned it from family members, including parents, grandparents, or even great-grandparents.

### 2.2. Medicinal Practices

Table 2 summarizes the medicinal uses of *T. ascendens* documented by seven key collaborators. This orchid is used to alleviate headaches, toothaches, stomach pain, menstrual discomfort, and general body aches, as well as to treat kidney disease and inflammation. Additionally, this orchid is used to address two cultural ailments known as “*mal de orin*” and “*sangre sucia*”; in this context, the collaborators regarded *T. ascendens* as a plant with blood-purifying properties.

Collaborators reported that the most used part of *T. ascendens* for remedies is the mature leaves, which are employed to treat all the documented health ailments except kidney disease, for which the root is used. For stomach pain and inflammation, a combination of roots and mature leaves can be prepared. Notably, collaborators refer to the roots as the new shoots of the plant, which correspond to the developing leaves.

The primary mode of preparing *T. ascendens* for medicinal use is an infusion, which is ingested as “*Agua de tiempo*,” which refers to a diluted infusion prepared for regular consumption as a beverage, applicable to all reported ailments except for toothache, where the preparation involves crushing the plant or part of it to extract a liquid, of which a drop is applied directly to the affected area. Collaborators emphasized that only a small piece of leaf should be used when making an infusion. They cautioned that the remedy is considered “hot,” and excessive consumption can exacerbate the discomfort. 

Regarding dosage, all collaborators agreed that the maximum recommended frequency for an infusion is twice a day for menstrual pain and three times a day for other ailments. The treatment should last three days, although it can be discontinued earlier if symptoms resolve.

## 3. Discussion

This study employed a key collaborator approach combined with the snowball sampling method to document the traditional use of *Trichocentrum ascendens* in the study area. Despite visiting twelve communities in the Chinantla region, only seven key collaborators were identified in five communities. In ethnobiological research, key knowledge holders are often few and socially recognized [32], particularly when traditional knowledge was acquired through oral transmission from family members. The small number of participants reflects the restricted distribution of medicinal *T. ascendens* knowledge within the study communities. Consequently, this knowledge constitutes part of a biocultural legacy that is at risk of disappearing. Therefore, the purposive recruitment of key collaborators prioritizes information quality and cultural validity over sample size [33].

The collaborators did not report any new medicinal uses for *T. ascendens* other than those documented in the first five interviews. This pattern is consistent with the study by Muellmann et al. [34], who compared the information obtained through the evaluation of the community preparation of 4 to 6 versus 6 to 15 key collaborators and found that thematic saturation was achieved after the fourth key collaborator; despite increasing the sample size, no additional information was obtained. Therefore, based on the present findings, thematic saturation has been achieved in the present study. On the other hand, 86% of respondents reported its analgesic effect in different diseases.

The medicinal uses for *T. ascendens* reported here suggest significant potential for future research focused on the discovery of new natural products and herbal remedies. Given its effectiveness in decreasing several types of pain, its application may be relevant for treating conditions involving inflammatory processes and nociception-related diseases. Local use of this orchid is also related to two cultural diseases called “*mal de orin*” and “*sangre sucia*”. In the communities studied, “*mal de orin*” is described as an illness characterized by pain, burning, or difficulty urinating; linked to “*orina caliente*” and “*sangre sucia*”, it is described as an accumulation of impurities in the blood associated with internal imbalance. On the other hand, from the Western biomedical perspective, the symptoms attributed to “*mal de orin*” are consistent with an inflammation of the bladder that results in pain when urinating [30], while “*sangre sucia*” is interpreted as a condition in which toxins accumulate in the blood [35]; therefore, blood purifiers are used for its treatment. These purifiers eliminate toxins and stimulate the liver, kidneys, or lymphatic system by neutralizing toxins or providing nutrients for proper blood function [36]. In this sense, *T. ascendens* is considered a plant for purifying the blood and treating urinary system problems.

The methods of using *T. ascendens* reported by collaborators in this study involve preparing infusions or crushed preparations with the mature or developing leaves. This preparation is similar to that of *Trichocentrum cebolleta*, another morphologically similar species within the same genus. The preparation and application of *T. cebolleta* also vary depending on the ailment: for abdominal cramps, a small piece of leaf is crushed in cold water; for heart pain, a leaf infusion is used [16]; and for bone fractures or severe pain, leaves are crushed with salt and applied externally [15]. This suggests that the anti-nociceptive compounds in these plants are thermostable, with heat enhancing their extraction.

On the other hand, *Trichocentrum brachyphyllum* is reported to be used as a hallucinogen in religious rituals and for analgesic properties, which might be linked to the presence of phenanthrenes or their derivatives, as well as alkaloids identified in this orchid. The medicinal preparation method involves crushing with salt, which may be linked to the presence of alkaloids [18]. Estivi et al. [37] propose that adding salt improves the extraction of alkaloids, supporting the validity of this traditional preparation method. It has been shown that these compounds, alkaloids [38,39] and phenanthrene [40,41], are known to exhibit antinociceptive and sedative biological activities. Similar effects have been reported in other orchids, such as *Laelia anceps* Lindl. and *Cyrtopodium macrobulbon* (Lex.) G.A. Romero and Carnevali, where the presence of the alkaloid oxymorphone is attributed to these properties [42]. In *Maxillaria densa* Lindl., the phenanthrene erianthridin has also been shown to produce antinociceptive effects [40]. To date, only a few pigments have been identified in *T. ascendens*, including carotenoids (neoxanthin, lutein, and chlorophyll) and xanthophylls (violaxanthin, antheraxanthin, and zeaxanthin) [31].

Despite the traditional medicinal uses of *T. ascendens* documented in this study, along with its historical medicinal use in Veracruz and Yucatan [1,14,26], no pharmacological studies have been conducted to evaluate its biological activity to date. The information provided by Cano-Asseleih et al. [28] regarding *T. ancendens* is misleading, as it attributes it to biological activities, such as inhibition of cancer cell line proliferation and induction of apoptosis, that do not correspond to this species. Instead, these activities are associated with a different but morphologically similar species, *Trichocentrum microchilum* (Bateman ex Lindl.) M.W. Chase N.H. Williams, as well as two other unrelated orchids, *Oncidium isthmi* Schltr. and *Myrmecophila humboldtii* (Rchb. f.) Rolfe.

Given the phylogenetic relationship among orchids known as “*cola de rata*” (*T. cebolleta*, *T. brachyphyllum*, and *T. ascendens*), it is plausible that *T. ascendens* also contains alkaloids and fenatrenos. However, to date, the identification has been limited to a few pigments; thus, it is crucial to completely identify the compounds in *T. ascendens* that may be responsible for their medicinal properties. Additionally, pharmacological studies are necessary to explore its potential, which could lead to the discovery of new drugs for the ailments documented here.

## 4. Materials and Methods

### 4.1. Study Zone

**Figure 2:**
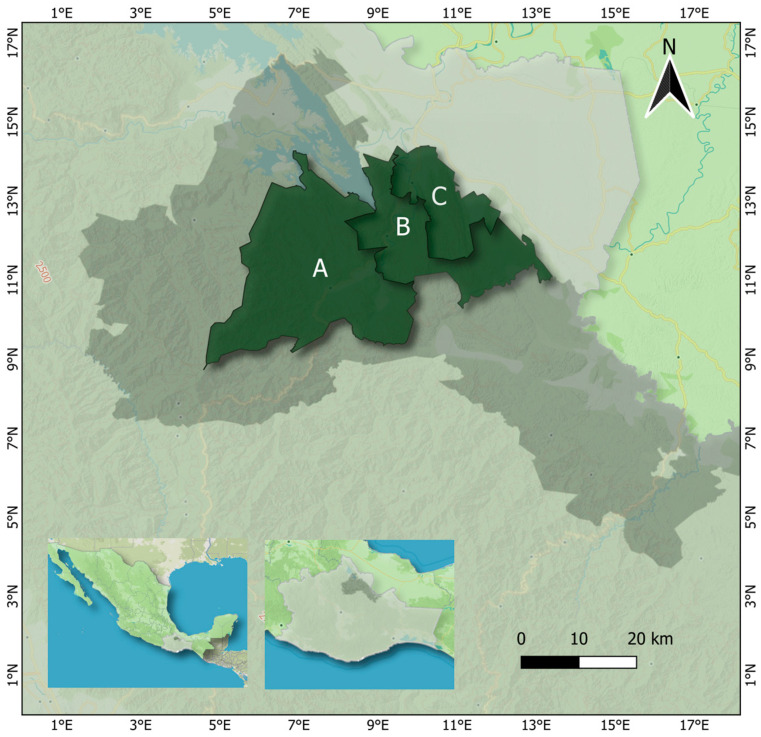
The geographic localization of the study zone in Chinantla region, Oaxaca, Mexico. Valle Nacional (A), Jacatepec (B) and Chiltepec (C).

The Chinantla region, characterized by complex topography and geomorphology [8], is situated in northeastern Oaxaca within the Papaloapan River Basin (Figure 2). It has been inhabited since pre-Hispanic times by the Chinanteco ethnic group, whose native language is Chinanteco [6]. Communities in this region experience high or extreme levels of marginalization and have low population density [43]. The region’s main economic activities include subsistence agriculture, livestock rearing [44], and multicropping staples such as corn, beans, and cassava. Shade-grown coffee [45] and vanilla [46] are cultivated for commercial purposes. Additionally, the fiber known as ixtle is produced from *Aechmea magdalenae* (André) André ex Baker [47]. Other activities involve the collection of palm and firewood for sale [48], raising of chickens and pigs [45], and hunting in certain areas [49].

To document the medicinal knowledge of *Trichocentrum ascendens* in the Chinantla region, twelve rural communities were visited. Key collaborators were identified only in the municipalities of San Juan Bautista Valle Nacional, Santa María Jacatepec, and San José Chiltepec (Figure 2). The selection of *T. ascendens* was based on preliminary information about its medicinal uses obtained from the local sources in Chinantla. These uses differ from those previously reported for the species in other regions of other states in Mexico, suggesting the locally specific knowledge and supporting its ethnopharmacological relevance.

### 4.2. Ethics Approval and Consent to Participate

The participants were verbally informed of the research’s purpose, and their consent was obtained for the publication of the information provided, ensuring the confidentiality of their identities at all times, following the guidelines of the Research Ethics Committee of the Instituto Nacional de Medicina, Ciencias y Nutrición Salvador Zubirán (INNSZ 2014). These guidelines align with the Declaration of Helsinki of the World Medical Association (WMA 2014).

### 4.3. Collection of Information

The communities shown on the map in Figure 2 were visited in May 2023. Semi-structured interviews were applied using the format outlined in Box 1 and the “snowball” method, which allows the identification and contact of hidden or hard-to-reach collaborators within a population [50]. The “key collaborators” approach was adopted, focusing on individuals with expertise in the medicinal flora of the region and specific medicinal uses of *T. ascendens*.

Box 1 **Interview format for collaborators on the medicinal uses of *Trichocentrum ascendens*.**

1) Name: __________________________                   2) Age: ___________________________

3) Occupation: _____________________                   4) Schooling: _______________________

5) Place of residence ________________________________________________________

6) Do you speak a native language? ___________________________________________

7) Which? __________________________________________________________________

8) Do you know the plant shown in the image?  Yes __ No ___

9) What is the name you know this plant by? ___________________________________

10) Do you know the uses of this plant? _______________________________________

11) Do you know or have you used this plant as a medicinal remedy? Yes __ No ___

12) For which ailments is the plant used? _______________________________________

13) What part of the plant is used? _____________________________________________

14) How do you prepare the remedy? _________________________________________

15) How do you take or apply the remedy? ____________________________________

16) Do you know of other similar plants that have medicinal use in your region? ____

17) Do you practice traditional medicine?

18) How many years have you practiced traditional medicine? ____________________

19) How did you acquire your knowledge about the use of plants in traditional medicine? ___

20) Do you belong to a society of traditional healers? Yes __ No ___

21) Which? ______________________________________________________________

Inclusion criteria required participants to be at least 18 years old, native to Chinantla, willing to provide informed consent, and recognized within their communities as knowledgeable about local plant use, including traditional healers, medicinal plant vendors, and farmers. Participants’ responses were documented verbatim in writing within the questionnaire during the interview sessions. 

The questionnaire aimed to document the following aspects: (i) the worldview associated with traditional knowledge and practices related to the orchid’s use; (ii) the socioeconomic characteristics of the individuals maintaining this knowledge, including age, gender, schooling, knowledge of the indigenous language, occupation, place of origin, and how the knowledge was acquired; and (iii) information on the diseases or conditions for which *T. ascendens* is used, part of the plant used, preparation methods, dosage, and characteristics of the individuals for whom it is recommended.

Before conducting the interviews, a specimen of *T. ascendens* was collected in the study area. Photographs of the entire plant and its floral structures were taken to prepare the illustration shown in Figure 1. This plate was used as a visual aid to ensure that the information provided by the participants accurately referred to *T. ascendens*. A voucher specimen identified by RS was herbarized and deposited in the OAX herbarium of the Instituto Politécnico Nacional (Solano 4430).

## 5. Conclusions

The state of Oaxaca is renowned for its rich biocultural heritage. However, the knowledge of indigenous peoples is vulnerable due to the migration of young people to other regions in search of greater purchasing power. In the Chinantla region of Oaxaca, *T. ascendens* is traditionally used to relieve headaches, toothaches, stomachaches, and menstrual and bodily discomfort, as well as to treat general inflammation and kidney conditions, including urinary tract infections and diseases culturally defined as “mal de orín” and “*sangre sucia*.” Medicinal preparations consist of infusions or crushed leaves, administered for short periods (three days). The limited number of key collaborators in the study area suggests that knowledge about *T. ascendens* is unevenly distributed and potentially at risk. The results of this research confirm the importance of documenting these traditional practices to preserve biocultural heritage.

## Figures and Tables

**Table 1:** Socioeconomic data of collaborators knowledgeable about the medicinal use of *Trichocentrum ascendens*. Gender: F, female; M, male. Schooling: PES, primary elementary school; SES, secondary elementary school; HS, high school; UG, undergraduate students; NS, no schooling. Language: S, Spanish; Bl, bilingual (Chinantec and Spanish).

Informant	Gender	Age	Occupation	Education	Place of Residence	Language	Medicinal Use	Knowledge Source
1	F	29	Medicinal plant traders	HS	Santa María Jacatepec	S	Stomachache, headache, swelling, coughing	Traditional medicine practicioner
2	M	42	Farmer	UG	La Gran Lucha, Valle Nacional	Bl	Headache and toothache	Father
3	M	58	Traditional healer	NS	San José Chiltepec	Bl	Kidney disease	Great-grandfather
4	F	31	Traditional healer	UG	Ejido Emiliano Zapata, Santa María Jacatepec	Bl	“*Mal de orin*” and “*sangre sucia*”	Mother
5	F	59	Traditional healer	PES	Ejido Emiliano Zapata, Santa María Jacatepec	Bl	“*Mal de orin*” and “*sangre sucia*”, painful menstruation	Great aunt
6	M	37	Farmer	HS	Plan de Águila, San José Chiltepec	Bl	Headache	Family
7	F	32	Seasonal vendor ^1^	UG	Plan de Águila, San José Chiltepec	S	Headache and body pain	Family

Note: ^1^ Seasonal vendor refers to an individual who seasonally sells artisanal and natural products at cultural and gastronomic fairs across different regions of the state.

**Table 2:** Summary of medicinal uses of the orchid *Trichocentrum ascendens* in the studied zone.

Condition	Plant Part Used	Preparation	Dosage	Patient Type
Stomachache	Leaves	Infusion	“*Agua de tiempo*” max. 3 days (small leaf piece)	All ages
Headache	Leaves	Infusion	“*Agua de tiempo*” max. 3 days (small leaf piece)	All ages
Menstrual pain	Leaves	Infusion	“*Agua de tiempo*”; 2×/day, max. 3 days (small leaf piece)	Women
Toothache	Leaves	Crushed	A drop of the crushed leaves, when the pain occurs	All ages
Body pain	Leaves	Infusion	“*Agua de tiempo*”; max. 3 days (small leaf piece)	All ages
Inflammation	Leaves	Infusion	3×/day, max. 3 days (small leaf piece)	All ages
Kidney diseases	Leaves	Infusion	3×/day, max. 3 days (small leaf piece)	All ages
“*Mal de orin*”	Leaves	Infusion	3×/day, max. 3 days (small leaf piece)	All ages
“*Sangre sucia*”	Leaves	Infusion	3×/day, max. 3 days (small leaf piece)	All ages
Cough	Leaves	Infusion	“*Agua de tiempo*” max. 3 days (small leaf piece)	All ages

## Data Availability

The original contributions presented in this study are included in the article. Further inquiries can be directed to the corresponding authors.
